# Softening of the Brain

**Published:** 1871-12

**Authors:** 


### SOFTENING OF THE BRAIN,

Called Ramollissement, is a second stage of inflammation of the brain, when its substance becomes infiltrated with blood and yellow matter. The delicate texture is broken down, softens, and runs together. The new-born infant, as well as the man of a hundred years, may have it. It is brought on alike by excessive action, or by none at all. The busiest brain and the laziest may alike be attacked with the fatal malady, for it is never cured. It comes on by the loss of use of some limb; sooner or later the mind does not work so freely; questions are answered slowly, and it is increasingly difficult to make one's self understood; by degrees the mental faculties become more and more impaired, the powers of sense more and more completely abolished, and finally all sense, all motion, is lost; he is but a breathing mass, and life goes out forever.

If a person has contraction of one or more limbs, steady and strong tingling and numbness at the end of the fingers, and an increasing forgetfulness and difficulty of conveying ideas by words, and perverted vision or blindness, then softening of the brain is imminent.

Professional men, active business men, especially those who eat a great deal and drink liquors largely, are the persons who are most likely to die of softening of the brain. It is almost impossible for any man who lives a sober, temperate life, combined with a moderate exercise of the brain and body, to have this most hopeless of all maladies.

